# Supplementary material for: Hard rocks and deep wetlands beneath Thwaites Glacier in Antarctica
Source: Commun Earth Environ. 2026 Apr 24;7(1):366. doi: 10.1038/s43247-026-03502-2 (PMC13109048; doi:10.1038/s43247-026-03502-2)
Supplement: Supplementary file 2 — Supplementary Information [file 43247_2026_3502_MOESM2_ESM.pdf]

Supplementary Information

Hard rocks and deep wetlands beneath Thwaites Glacier in Antarctica

Ole Zeising[1,⋆], Olaf Eisen[1,2], Coen Hofstede[1], Ronan Agnew[3], Alex Brisbourne[3], Andrew O. Hoffman[4,5], & Sridhar Anandakrishnan[6] on behalf of the GHOST Team*

[1]Glaciology, Alfred Wegener Institute Helmholtz Centre for Polar and Marine Research, Bremerhaven, Germany

[2]Faculty of Geosciences, University of Bremen, Bremen, Germany

[3]Ice Dynamics and Palaeoclimate, British Antarctic Survey, Natural Environment Research Council, Cambridge, UK

[4]Department of Earth, Environmental, and Planetary Science, Rice University, Houston, TX, USA

[5]Lamont-Doherty Earth Observatory, Columbia University, Palisades, NY, USA

[6]Department of Geosciences, and Earth and Environmental Systems Institute, Pennsylvania State University, University Park, PA, USA

*A full list of the consortium members appears at the end of the Supplementary Information

⋆corresponding author: ole.zeising@awi.de

This PDF file includes:

Supplementary Methods

SM1 Velocity analysis

SM1.1 Stacking velocity

SM1.2 Interval velocity

SM2 Amplitude analysis

SM2.1 Primary basal amplitude

SM2.2 Source amplitude

SM2.3 Attenuation

Supplementary Tables

Supplementary Table 1: Vibroseismic measurement meta data

Supplementary Table 2: Overview of subglacial media parameters

Supplementary Figures

Supplementary Figure 1: Region I and II of the along-flow profile

Supplementary Figure 2: Region III and IV of the along-flow profile

Supplementary Figure 3: Crag-and-tail in region III of the along-flow profile

Supplementary Figure 4: Region V and VI of the along-flow profile

Supplementary Figure 5: Region VII and VIII of the along-flow profile

Supplementary Figure 6: Region IX and X of the across-flow profile

Supplementary Figure 7: Region XI and XII of the across-flow profile

Supplementary Figure 8: Region XIII of the across-flow profile

Supplementary Figure 9: Geometry products along the extended along-flow profile

Supplementary Figure 10: Geometry products along the extended across-flow profile

Supplementary Figure 11: Bed classification of the along-flow and across-flow profiles

Supplementary Figure 12: Propagation velocity analysis of the along-flow profile

Supplementary Figure 13: Propagation velocity analysis of the across-flow profile

Supplementary Figure 14: Stacking velocity analysis at profile crossing

Supplementary Figure 15: Amplitude analysis of the basal return

Supplementary Figure 16: Amplitude analysis of the along-flow profile

Supplementary Figure 17: Amplitude analysis of the across-flow profile

Supplementary Figure 18: Quality factor of the along-flow profile

Members of participating consortium

Supplementary References

Supplementary Methods

SM1 Velocity analysis

SM1.1 Stacking velocity

The determination of the stacking velocity is a crucial component of the data processing procedures. We conducted the stacking velocity analysis using an automated constant velocity stacking method. We tested numerous NMO corrections for a broad range of velocities spanning from 3000 to $5000\,\mathrm{m\,s^{-1}}$ with $10\,\mathrm{m\,s^{-1}}$ intervals. We divided the TWT into segments of 20 ms with 10 ms overlap. For each segment, we identified the optimal stacking velocity based on the stack with the highest amplitude.

Next, we estimated the stacking velocities of the ice base, of the base of geological features beneath the ice, and of the bed. The stacking velocity at the ice base was calculated from the average of three TWT segments encompassing the ice base. A similar procedure was applied for the geological features. To determine the stacking velocity of the bed, we averaged the velocities of five TWT segments situated approximately 250 ms beneath the ice base or beneath geological features, respectively. For ice and bed, we averaged the stacking velocities over a wide range of 500 CMP gathers and for geological features over 50 CMP gathers.

Supplementary Figure 14 shows the stacking velocity analysis of the ice base derived from CMP gathers at the crossing of the two profiles in along- and across-flow direction. The derived stacking velocity from the CMP gather of the along-flow profile is $v_{\mathrm{stk}} = 3760\,\mathrm{m\,s^{-1}}$. Conversely, the obtained stacking velocity at the same CMP from the across-flow profile is $v_{\mathrm{stk}} = 3920\,\mathrm{m\,s^{-1}}$, indicating a difference of $160\,\mathrm{m\,s^{-1}}$. However, the basal return is found in both stacked traces at the same TWT, suggesting that the p-wave velocity in the vertical direction is consistent. This finding indicates that anisotropy exerts a significant influence on the horizontal component of the propagation velocity.

Supplementary Figures 12 and 13 show the velocity analysis of the two profiles in along-flow and across-flow direction. The average stacking velocity for the along-flow profile, is $3780 \pm 30\,\mathrm{m\,s^{-1}}$ (mean $\pm$ standard deviation) and exhibits no trend along the profile (Supplementary Figure 12b). In contrast, the average stacking velocity of the across-flow profile, is $3900 \pm 100\,\mathrm{m\,s^{-1}}$ (Supplementary Figure 13b), and thus significantly higher. Near the eastern shear margin of Thwaites Glacier, the estimated stacking velocity reaches its maximum of $4130\,\mathrm{m\,s^{-1}}$. Inside the shear margin, the velocity significantly decreases and reaches low values of $3621\,\mathrm{m\,s^{-1}}$ outside the glacier.

SM1.2 Interval velocity

Due to the ice crystal anisotropy, which influences stacking velocities, it is not possible to directly translate stacking velocities to ice interval velocities. To align with airborne radar-derived ice thicknesses, we estimated the interval velocities to be approximately $3770 \pm 35\,\mathrm{m\,s^{-1}}$ (along-flow profile, Supplementary Figure 12c) and $3770 \pm 50\,\mathrm{m\,s^{-1}}$ (across-flow profile, Supplementary Figure 13c). The interval velocities of sedimentary basins, tails of crag-and-tails and transparent zones were determined based on the Dix-Dürbaum-Krey equation:

$$v_n^2 = \frac{(v_{\mathrm{stk}}^n)^2 t_0^n - (v_{\mathrm{stk}}^{n-1})^2 t_0^{n-1}}{t_0^n - t_0^{n-1}} \tag{1}$$

where v_n represents the interval velocity of the n-th layer, v_{stk} denotes the stacking velocity of the n-th and $n-1$-th layers, and their respective zero-offset travel times t_0. We estimated the interval velocity for a large sedimentary basin to be $2680 \pm 440\,\mathrm{m\,s^{-1}}$ (along-flow profile, Supplementary Figure 12c) and for the transparent features to be $1960 \pm 420\,\mathrm{m\,s^{-1}}$ (across-flow, Supplementary Figure 13c) We assumed a constant interval velocity of the bed of $4000\,\mathrm{m\,s^{-1}}$. These interval velocities were used in the processing for migration and depth conversion.

SM2 Amplitude analysis

SM2.1 Primary basal amplitude

We used an algorithm to determine the amplitude A_1 of the prime basal reflection for small offsets from CMP gathers. First, we identified A_1 candidates (local maxima, $l_{\mathrm{max},3}$) that were larger than 50% of the maximum amplitude in a window of -40 to $+10$ ms around the ice base. Next, we formulated four criteria to determine A_1 based on the characteristics of a Ricker wavelet as observed with the direct wave. These criteria include the two local maxima, namely $l_{\mathrm{max},1}$ and $l_{\mathrm{max},2}$, which are positioned above $l_{\mathrm{max},3}$, and $l_{\mathrm{max},4}$ situated below $l_{\mathrm{max},3}$:

1. The candidate $l_{\mathrm{max},3}$ has the opposite polarity to the local maximum above and below:
   $l_{\mathrm{max},2} \times l_{\mathrm{max},3} < 0$ and $l_{\mathrm{max},3} \times l_{\mathrm{max},4} < 0$

2. The absolute amplitudes of $l_{\mathrm{max},1}$, $l_{\mathrm{max},2}$ and $l_{\mathrm{max},3}$ increase with depth:
   $|l_{\mathrm{max},1}| < |l_{\mathrm{max},2}| < |l_{\mathrm{max},3}|$.

3. The absolute amplitude of $l_{\mathrm{max},3}$ is larger than $l_{\mathrm{max},4}$:
   $|l_{\mathrm{max},3}| > |l_{\mathrm{max},4}|$.

4. The absolute amplitude of $l_{\max,4}$ is larger than $l_{\max,2}$:
   $|l_{\max,4}| > |l_{\max,2}|$.

5. The increase in absolute amplitude from $l_{\max,2}$ to $l_{\max,3}$ is significant:
   $(|l_{\max,2}| - |l_{\max,1}|)/2 < (|l_{\max,3}| - |l_{\max,2}|)$.

We defined A_1 as the shallowest candidate that satisfied at least four of these five criteria (Supplementary Figure 15).

SM2.2 Source amplitude

To determine the source amplitude, we utilized the multiple bounce method [1]. This method derives the source amplitude from the amplitude decay between the primary (single bounce amplitude A_1) and first multiple reflections (double bounce amplitude A_2):

$$A_0 = -\frac{A_1^2}{A_2}\frac{1}{2\gamma_1}. \tag{2}$$

For this purpose, we selected 21 shot gathers with a pronounced multiple reflection of the ice base in 7 areas distributed along both profiles (Supplementary Figures 16, 17). From these, we extracted the amplitudes A_1 and A_2 for small offsets $< 750\,\mathrm{m}$ (angle of incidence $\theta < 10°$), and subsequently determined the range of A_0 (Supplementary Figures 16c, 17c). After removing outliers, the average source amplitude $\overline{A}_0$ was determined to be $0.8 \pm 0.4 \times 10^{11}$.

To assess the consistency of the source power, we calculate the amplitude of the direct wave (A_D) for all shots and offsets. For each offset, we determined the relative deviation from the mean across all shots ($A_D/\overline{A_D}$). We then smoothed the amplitudes based on a running mean with a window of 3 km and averaged the relative deviation across all offsets. The results demonstrate small deviations on smaller scales, but indicates trends on larger scales (Supplementary Figures 16c, 17c). Deviations from the mean of up to 50% are consistent with the observed variability of $\overline{A}_0$. Consequently, in further processing we use a relative power dependent source amplitude A_0:

$$A_0 = \overline{A}_0\frac{A_D}{\overline{A_D}}. \tag{3}$$

The direct wave amplitude of the along-flow profile exhibits a trend from lower amplitudes in the downstream region to higher amplitudes upstream. This trend may be attributed to changes in surface conditions with increasing surface elevation from 810 to 1360 m (Supplementary Figure 16a).

SM2.3 Attenuation

To estimate the basal reflection coefficient from the observed amplitudes, we must account for power loss due to seismic wave attenuation within the ice. The attenuation α can be quantified using the quality factor Q, which describes the efficiency with which a material stores and dissipates elastic energy:

$$\alpha = \frac{\pi f}{Qv} \tag{4}$$

where f is the frequency of the seismic wave, and v is the seismic wave velocity.

To estimate Q, we use the frequency-dependent amplitude decay between the prime A_p and multiple A_m basal reflection, following the spectral ratio method:

$$\ln(A_m(f) - \ln(A_p(f)) = -\frac{\pi \Delta t}{Q} f + \text{constant} \tag{5}$$

[2, 3], where Δt is the travel time difference between the prime and the multiple basal return. Q is obtained by a linear regression of $\ln(A_m(f)) - \ln(A_p(f))$ against frequency, with a gradient of $-\pi\Delta t/Q$. First, we selected 75 shots of along-flow profile with a strong multiple and stacked the traces or the prime and multiple after windowing with a Blackman-Harris window (Supplementary Figure 18a,b). We then computed the frequency spectra of the stacked primary and multiple reflections (Supplementary Figure 18c,d) and subtracted their natural logarithms to obtain the log spectral ratio: $\ln(A_m(f)) - \ln(A_p(f))$ (Supplementary Figure 18e). Next, we calculated the gradient of the log spectral ratio over the upper $\sim 40\%$ frequency range of the sweep after filtering (110–190 Hz) and obtained $Q = 451 \pm 23$. To estimate the attenuation, we used the centroid frequency of the power spectrum of $f = 146 \pm 25\,\mathrm{Hz}$ and the interval velocity of ice $v_{\mathrm{ice}} = 3770\,\mathrm{m\,s^{-1}}$, giving an estimated attenuation of $\alpha = (2.7 \pm 0.5) \times 10^{-4}\,\mathrm{m^{-1}}$.

Supplementary Tables

Supplementary Table 1: **Vibroseismic measurement meta data.** Setup and acquisition parameters of the two along- and across-flow profiles.

<table>
<tr><th></th><th>Along-flow profile</th><th>Across-flow profile</th></tr>
<tr><td>Profile number</td><td>20230551</td><td>20240551</td></tr>
<tr><td>Start date</td><td>31 Dec 2022</td><td>31 Dec 2023</td></tr>
<tr><td>End date</td><td>13 Jan 2023</td><td>09 Jan 2024</td></tr>
<tr><td>Start coordinates</td><td>75.98° S, 107.56° W</td><td>76.44° S, 108.06° W</td></tr>
<tr><td>End coordinates</td><td>77.83° S, 109.24° W</td><td>76.35° S, 102.99° W</td></tr>
<tr><td>Profile length</td><td>210 km</td><td>134 km</td></tr>
<tr><td>Number of CMPs</td><td>16822</td><td>10729</td></tr>
<tr><td>Sweep frequencies</td><td>10 – 250 Hz</td><td>10 – 200 Hz</td></tr>
<tr><td>Sweep length</td><td colspan="2">10 s</td></tr>
<tr><td>Taper length</td><td colspan="2">0.5 s</td></tr>
<tr><td>Peak Force</td><td colspan="2">60 %, 40 kN</td></tr>
<tr><td>Shot interval</td><td colspan="2">75 m</td></tr>
<tr><td>Group interval</td><td colspan="2">25 m</td></tr>
<tr><td>Number of receiver groups</td><td colspan="2">60</td></tr>
<tr><td>Minimum offset</td><td colspan="2">43 m</td></tr>
<tr><td>Maximum offset</td><td colspan="2">1518 m</td></tr>
<tr><td>CMP interval</td><td colspan="2">12.5 m</td></tr>
<tr><td>Fold of coverage</td><td colspan="2">10</td></tr>
<tr><td>Sampling interval</td><td colspan="2">0.5 ms</td></tr>
<tr><td>Maximum time</td><td colspan="2">4 s</td></tr>
</table>

Supplementary Table 2: **Overview of subglacial media parameters.** P-wave velocities V_p, densities ρ, reflection coefficients R_1 and acoustic impedance Z_b for subglacial media at normal incidence [4].

Lithology / hydrology	V_p ($\mathrm{m\,s^{-1}}$)	ρ ($\mathrm{kg\,m^{-3}}$)	R_1	Z_b ($\mathrm{kg\,m^{-2}\,s^{-1}}$)
Lithified sediments/ Crystalline basement	3000 – 6200	2200 – 2800	0.33 – 0.68	6.60×10^6 – 17.36×10^6
Consolidated sediments	2000 – 2600	1600 – 1900	−0.02 – 0.20	3.20×10^6 – 4.94×10^6
Unconsolidated sediments	1700 – 1900	1600 – 1800	−0.10 – 0.01	2.72×10^6 – 3.42×10^6
Dilatant till	1600 – 1800	1600 – 1800	−0.13 – −0.01	2.56×10^6 – 3.24×10^6
Water	1450 – 1498	1000 – 1020	−0.39 – −0.37	1.45×10^6 – 1.53×10^6

Supplementary Table 3: **Overview of bed classification.** Basal conditions are classified into three categories (soft, intermediate, and hard bed) based on inferred resistance to deformation, using acoustic impedance (Z_b) as a proxy for ice–bed interaction. Impedance thresholds are defined by the overlapping ranges of dilatant till and unconsolidated sediments. The intermediate bed class represents a mechanically unresolved basal regime arising from limited sensitivity of small-offset seismic measurements.

Bed class	Ice–bed interaction	Z_b ($\mathrm{kg\,m^{-2}\,s^{-1}}$)
Hard bed	Sliding–dominated basal motion	$> 3.24 \times 10^6$
Intermediate bed	Ambiguous deformation regime	2.72×10^6 – 3.24×10^6
Soft bed	Till-deformation–dominated basal motion	$< 2.72 \times 10^6$

105 # Supplementary Figures

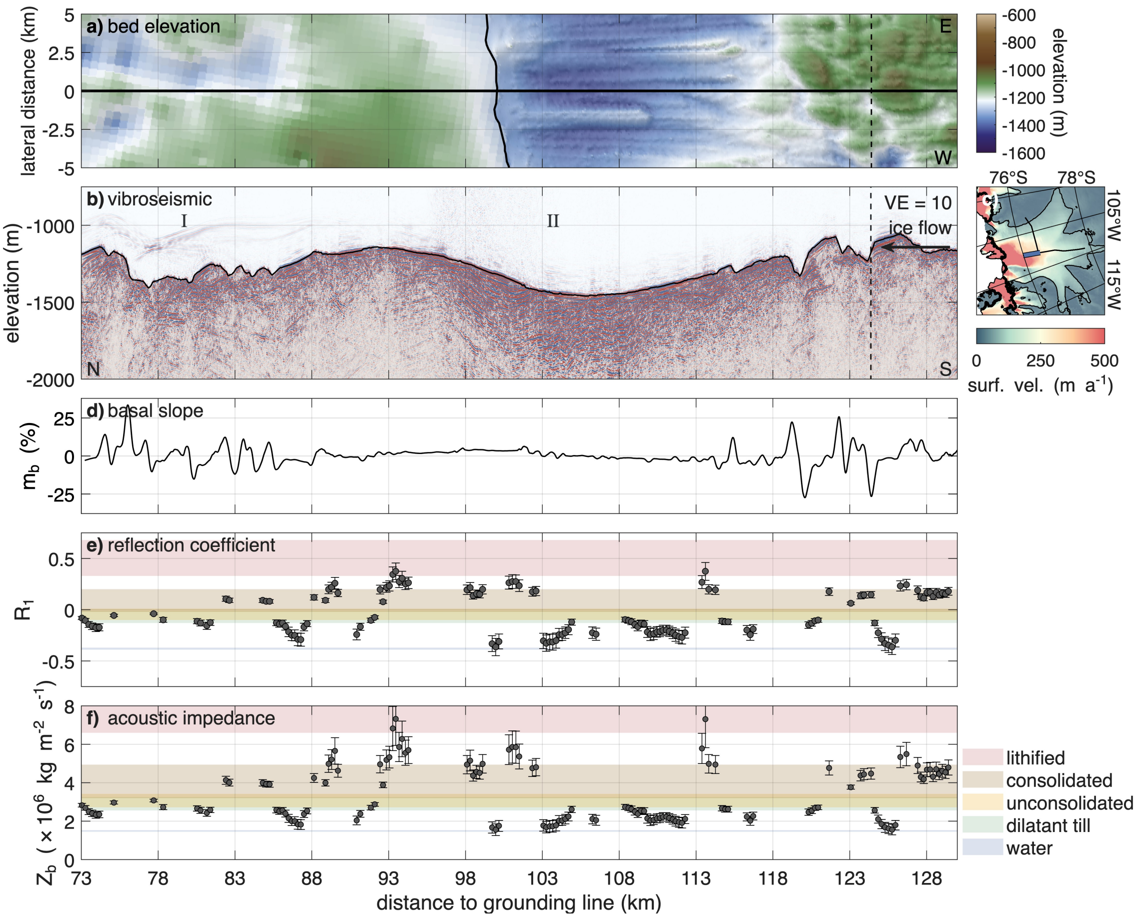

Supplementary Figure 1: **Region I and II of the along-flow profile.** **a** Map view of bed elevation (WGS 84) from Bedmap3 [5, 6] and swath airborne radar [7] in lateral direction. **b** Kirchhoff poststack time migrated vibroseismic section with a vertical exaggeration VE = 10. **c** Map of Thwaites Glacier with location of vibroseismic section (blue line). Details are presented in Fig. 1e. **d** Basal slope m_b in ice flow direction, calculated from a mean gradient over twice the Fresnel zone radius ($\sim 220\,$m). **e** Basal reflection coefficient R_1. **f** Basal acoustic impedance Z_b. The shown profiles are a function of distance to grounding line. Ice flow from right to left. Error bars indicate uncertainties in the reflection coefficient and acoustic impedance, as described in the Methods.

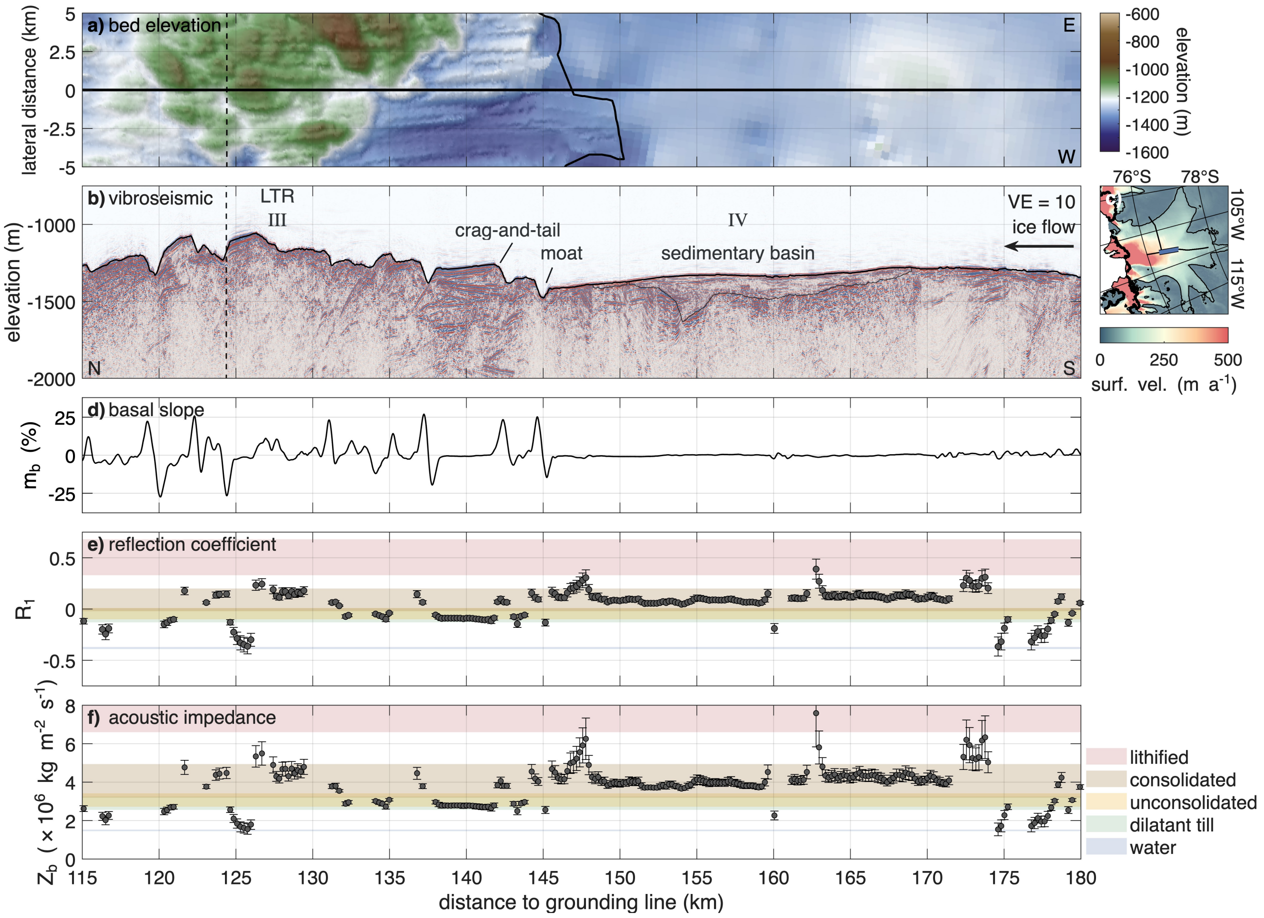

Supplementary Figure 2: **Region III and IV of the along-flow profile.** Figure panels and variables are identical to those described in Supplementary Figure 1.

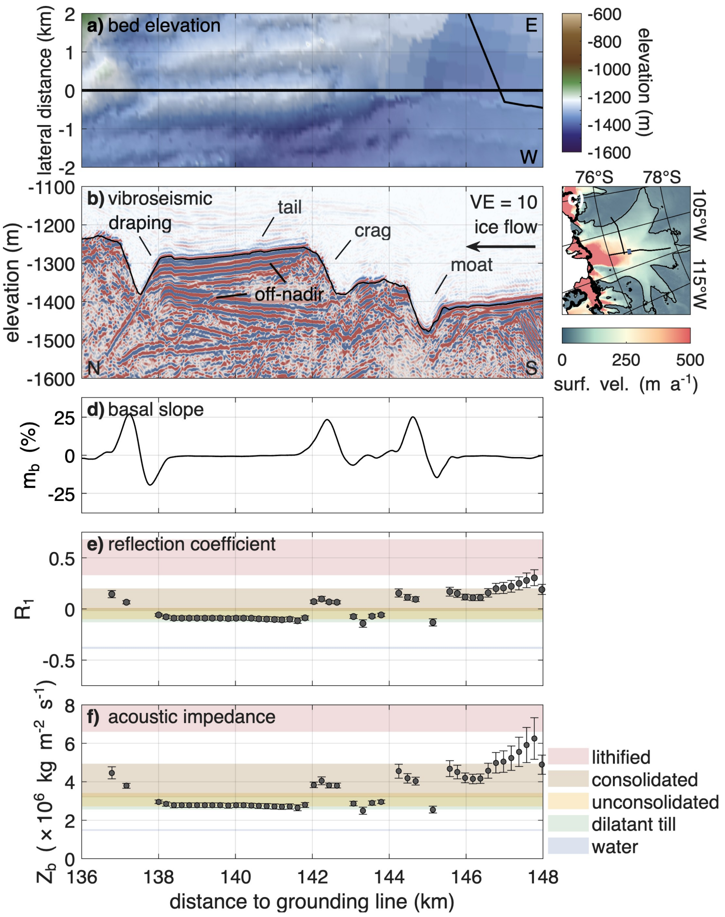

Supplementary Figure 3: **Crag-and-tail in region III of the along-flow profile.** Figure panels and variables are identical to those described in Supplementary Figure 1.

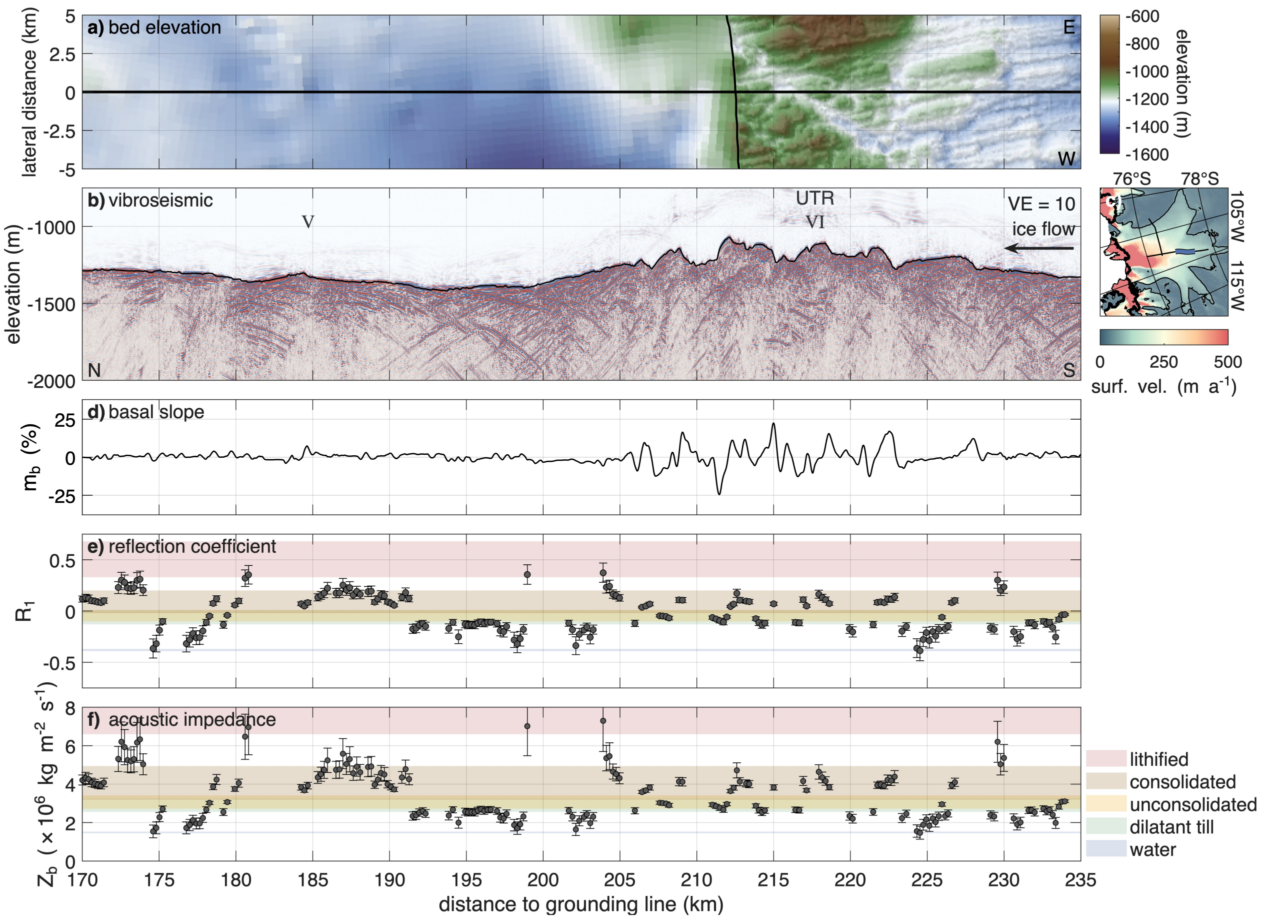

Supplementary Figure 4: **Region V and VI of the along-flow profile.** Figure panels and variables are identical to those described in Supplementary Figure 1.

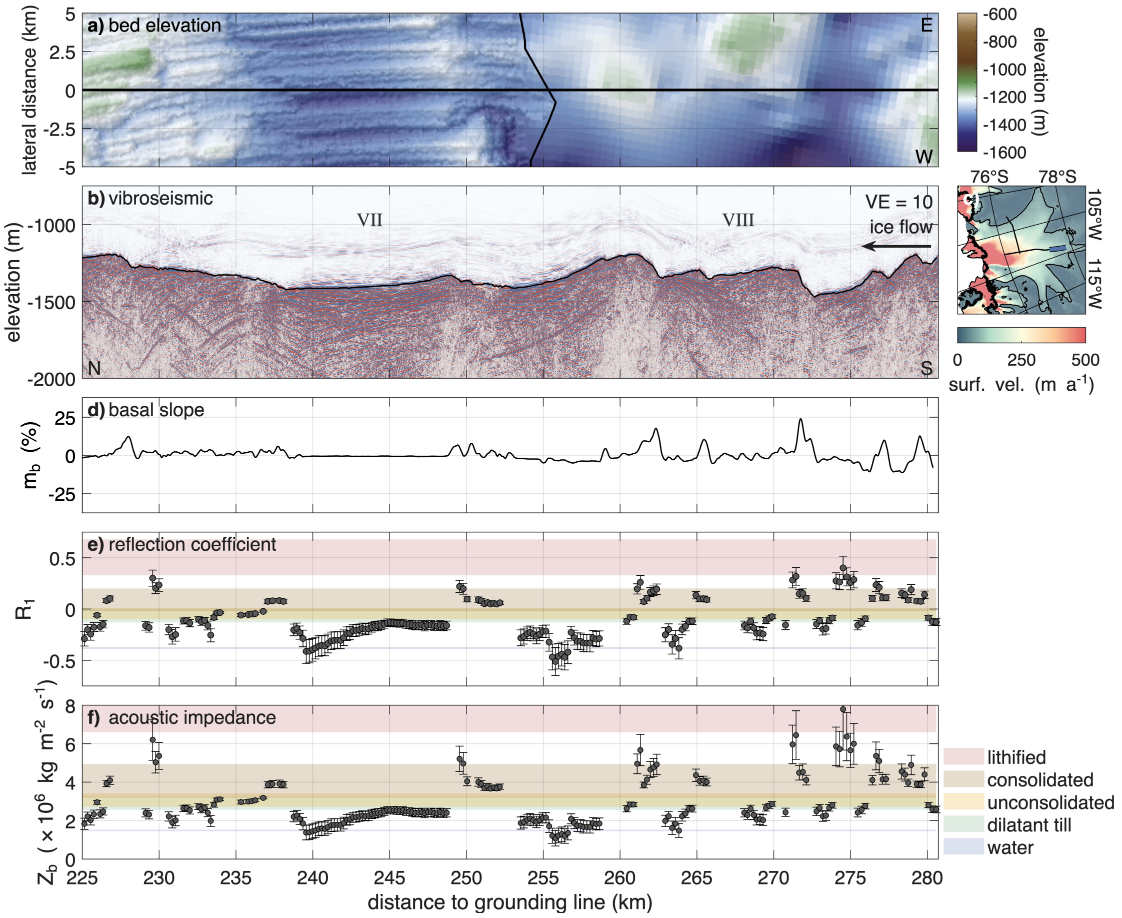

Supplementary Figure 5: **Region VII and VIII of the along-flow profile.** Figure panels and variables are identical to those described in Supplementary Figure 1.

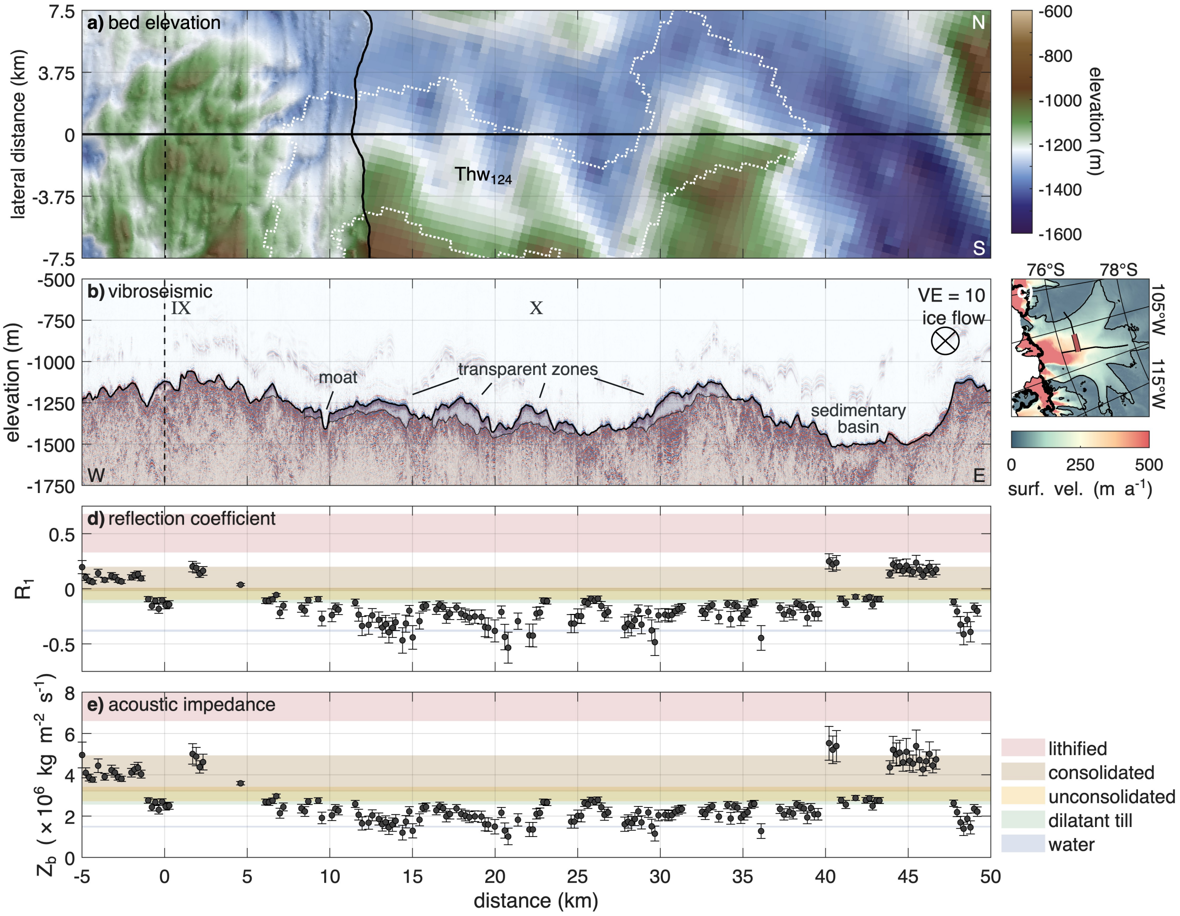

Supplementary Figure 6: **Region IX and X of the across-flow profile.** **a** Map view of bed elevation (WGS 84) from Bedmap3 [5, 6] and swath airborne radar [7] in lateral direction. The dashed line indicates the intersection with the along-flow profile. The white, dotted line marks the outline of a subglacial lake, derived from the drainage between 2022 and 2024. **b** Kirchhoff migrated vibroseismic section with a vertical exaggeration VE = 10. **c** Map of Thwaites Glacier with location of vibroseismic section (red line). Details are presented in Fig. 1e. **d** Basal reflection coefficient R_1. **e** Basal acoustic impedance Z_b. The shown profiles are a function of distance to the along-flow profile. Ice flow into the page. Error bars indicate uncertainties in the reflection coefficient and acoustic impedance, as described in the Methods.

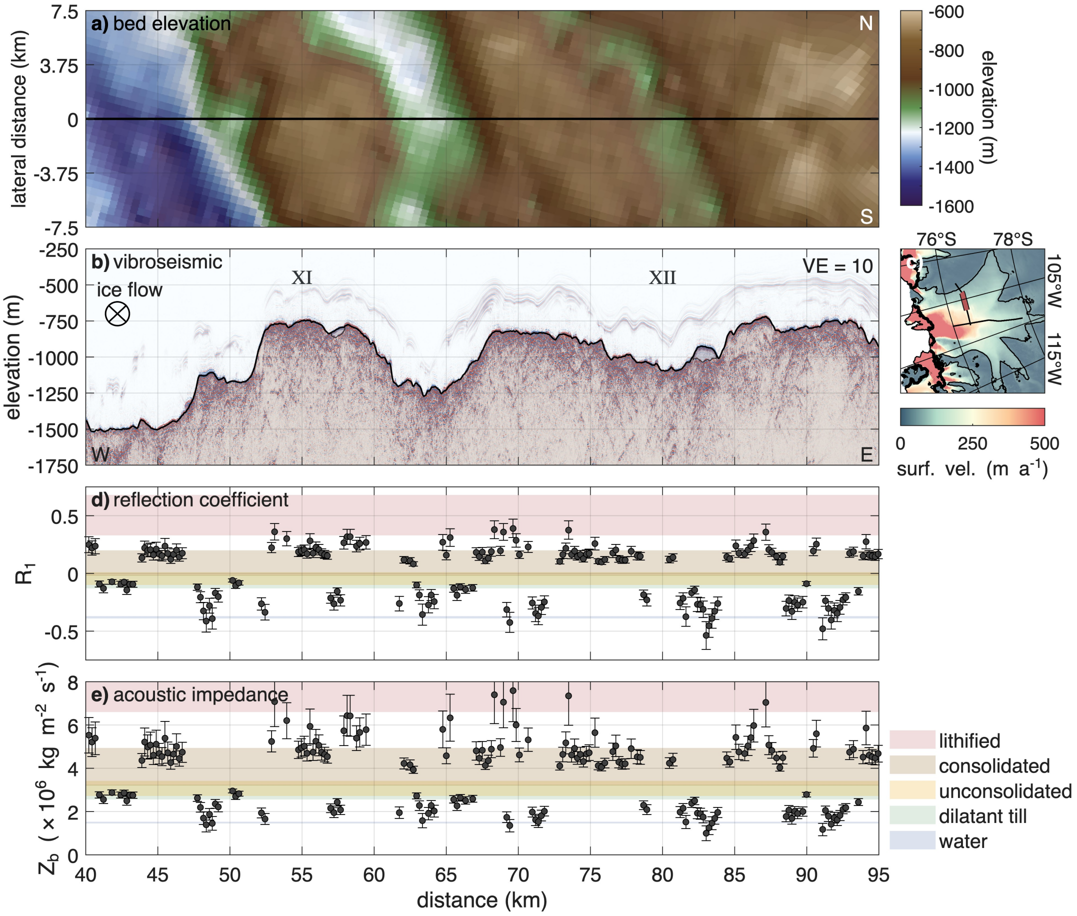

Supplementary Figure 7: **Region XI and XII of the across-flow profile.** Figure panels and variables are identical to those described in Supplementary Figure 5.

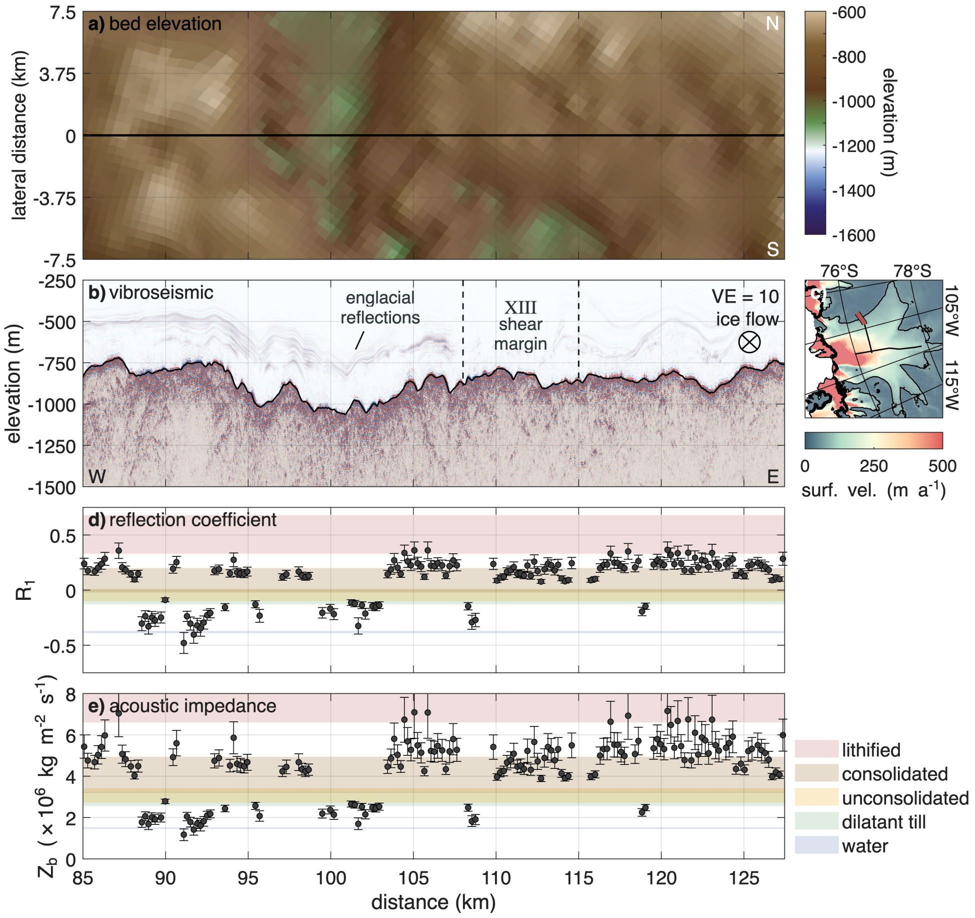

Supplementary Figure 8: **Region XIII of the across-flow profile.** Figure panels and variables are identical to those described in Supplementary Figure 5.

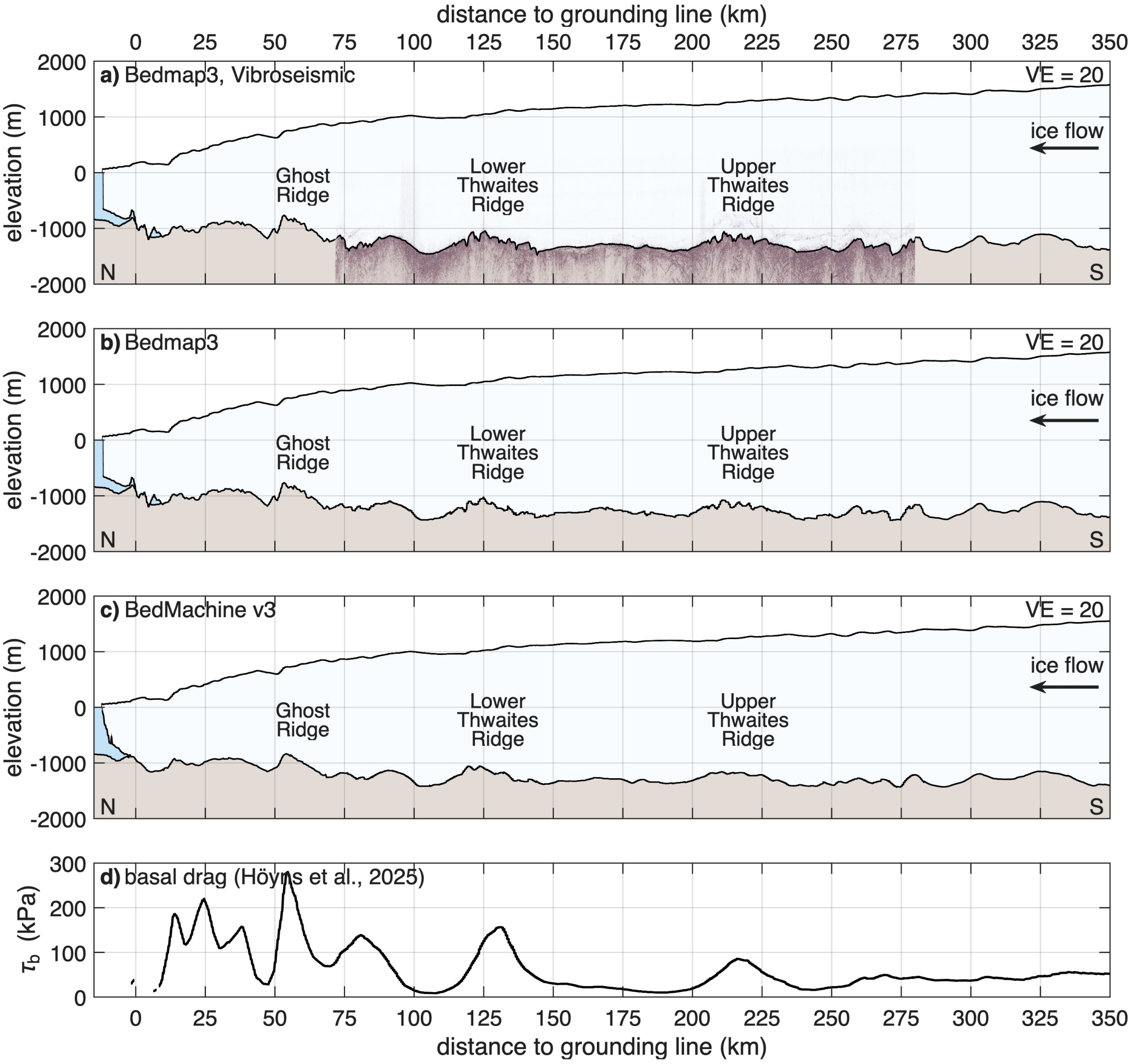

Supplementary Figure 9: **Geometry products along the extended along-flow profile. a** Bedmap3 [5, 6] with migrated vibroseismic profile and in **b** without vibroseismic profile. **c** BedMachine Antarctica v3 [8, 9]. Elevation relative to WGS 84. The sections are vertically exaggerated by a factor of VE=20. **d** Basal drag derived for a sliding exponent of $m = 3$ and a Budd-type friction law [10].

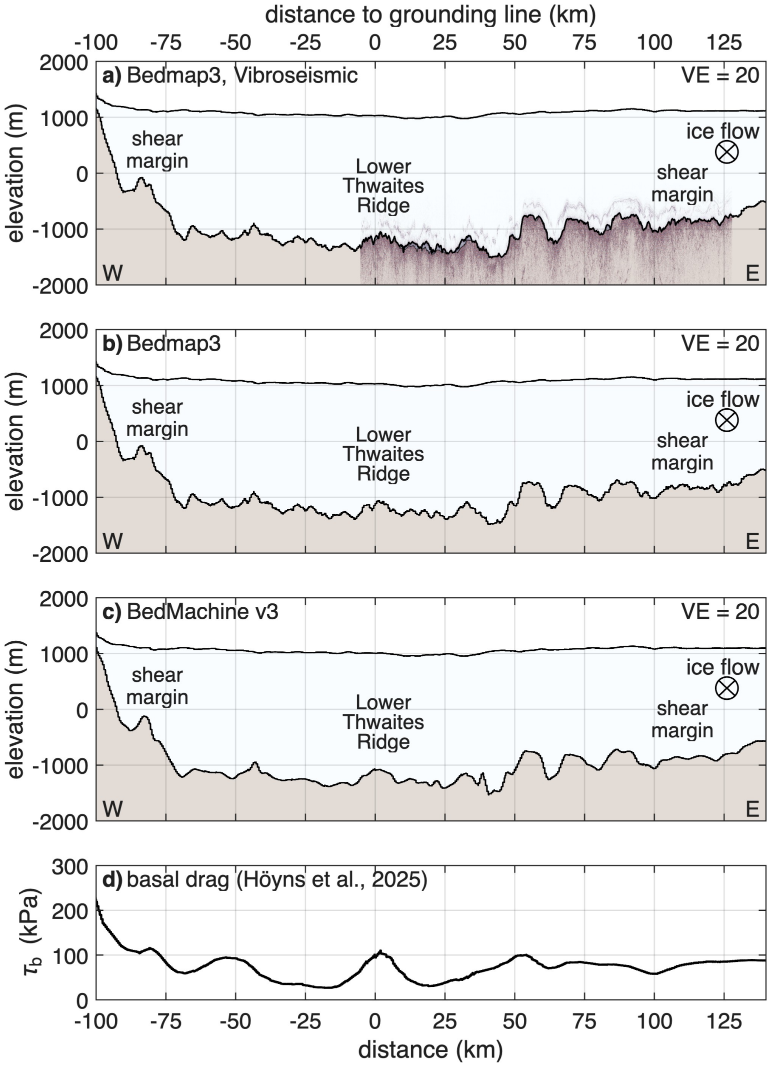

Supplementary Figure 10: **Geometry products along the extended across-flow profile.** **a** Bedmap3 [5, 6] with migrated vibroseismic profile and in **b** without vibroseismic profile. **c** BedMachine Antarctica v3 [8, 9]. Elevation relative to WGS 84. The sections are vertically exaggerated by a factor of VE=20. **d** Basal drag derived for a sliding exponent of $m = 3$ and a Budd-type friction law [10].

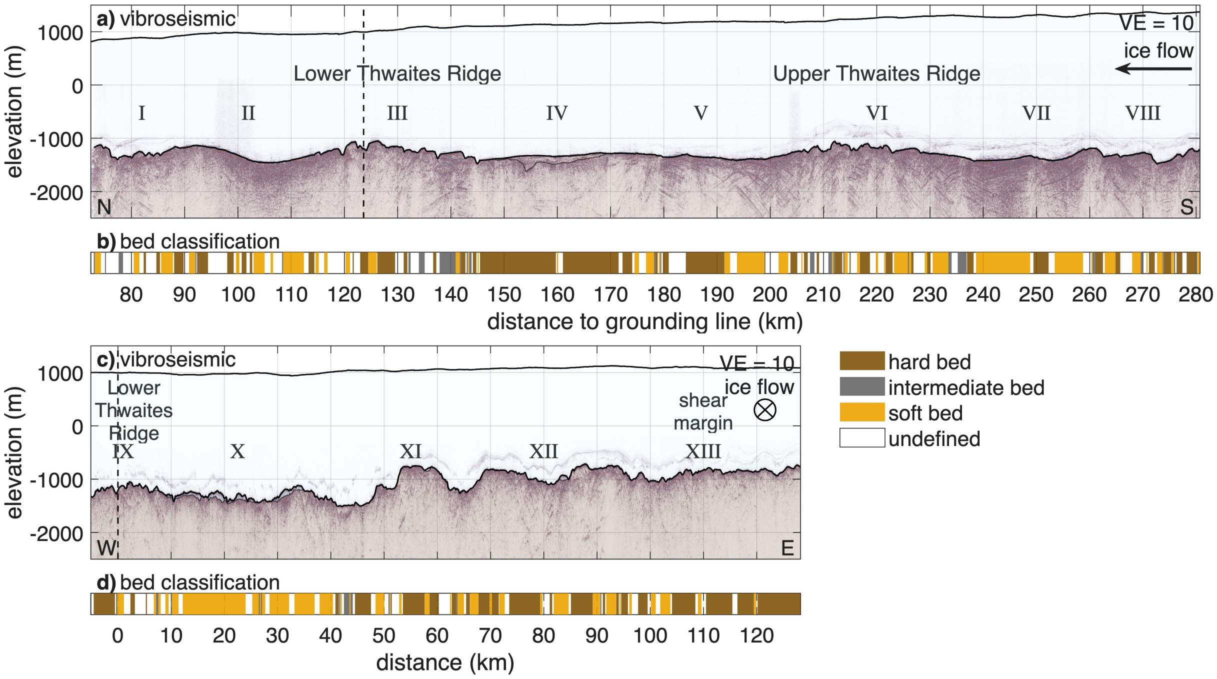

Supplementary Figure 11: **Bed classification of the along-flow and across-flow profiles.** **a, c** Kirchhoff migrated vibroseismic profiles in along-flow (a) and across-flow (c) direction with a vertical exaggeration VE = 10. **b, d** Classification of basal conditions into hard ($Z_b > 3.24 \times 10^6\,\mathrm{kg\,m^{-2}\,s^{-1}}$), intermediate ($2.72 \times 10^6 < Z_b < 3.24 \times 10^6\,\mathrm{kg\,m^{-2}\,s^{-1}}$), and soft beds ($Z_b < 2.72 \times 10^6\,\mathrm{kg\,m^{-2}\,s^{-1}}$).

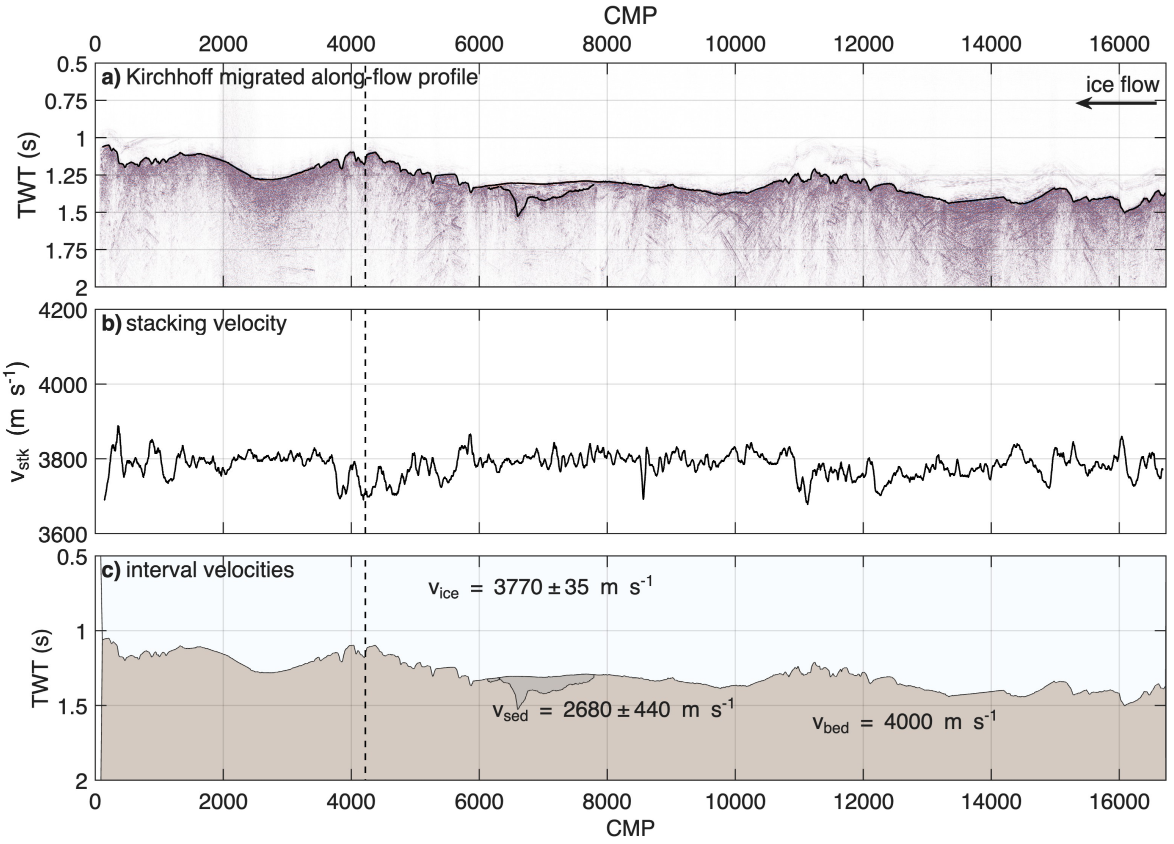

Supplementary Figure 12: **Propagation velocity analysis of the along-flow profile.** **a** Kirchhoff migrated vibroseismic section as a function of TWT and CMP. **b** Algorithm detected p-wave ice velocities from NMO analysis. **c** Interval velocities of ice (v_{ice}), sediment basin (v_{sed}) and bed (v_{bed}) used for migration and depth conversion. The vertical dashed line marks the crossing of the profiles.

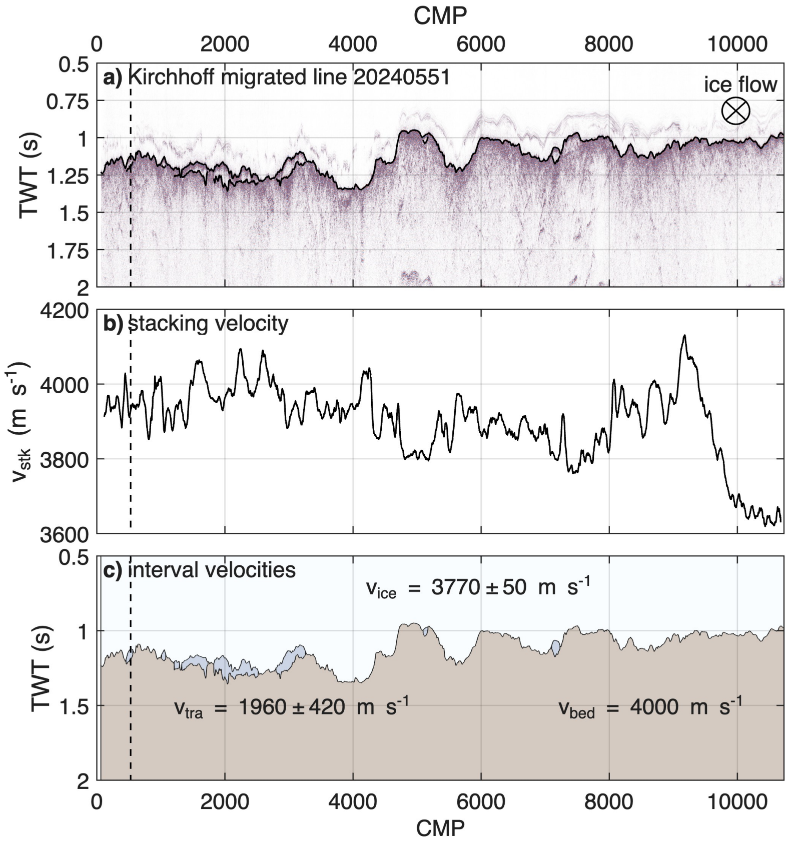

Supplementary Figure 13: **Propagation velocity analysis of the across-flow profile. a** Kirchhoff migrated vibroseismic section as a function of TWT and CMP. **b** Algorithm detected p-wave ice velocities from NMO analysis. **c** Interval velocities of ice (v_{ice}), transparent features (v_{tra}) and bed (v_{bed}) used for migration and depth conversion. The vertical dashed line marks the crossing of the profiles.

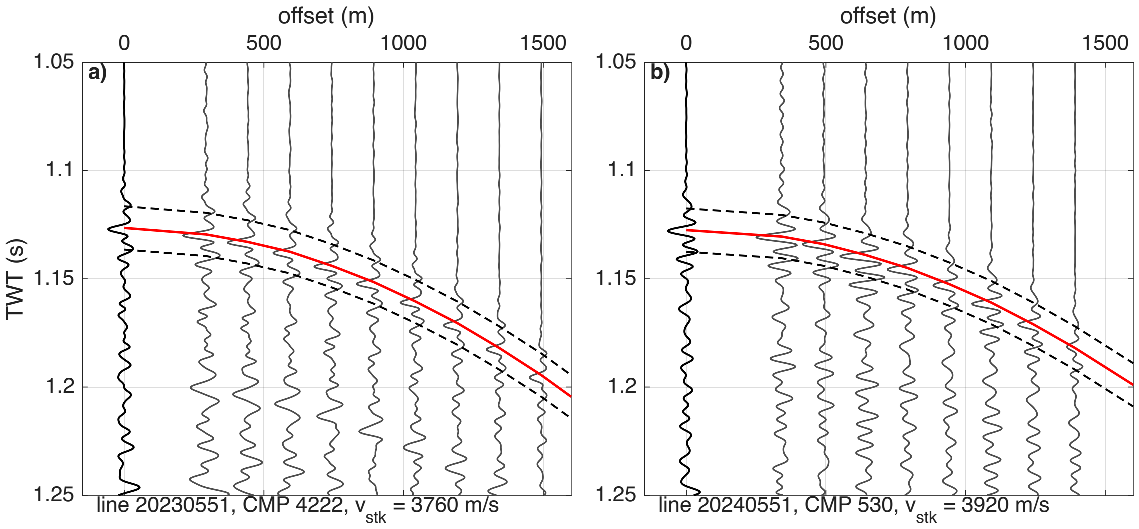

Supplementary Figure 14: **Stacking velocity analysis at profile crossing.** **a** CMP 4222 of along-flow profile 20230551. The stacked trace at zero offset was derived after a NMO correction with a velocity of 3760 m s^{-1}. **b** CMP 530 of across-flow profile 20240551. The stacked trace at zero offset was derived after a NMO correction with a velocity of 3920 m s^{-1}. The red line represents the hyperbola corresponding to the specified stacking velocity. The two dashed lines delineate the boundaries of the 20 ms segment used to identified the stacking velocity.

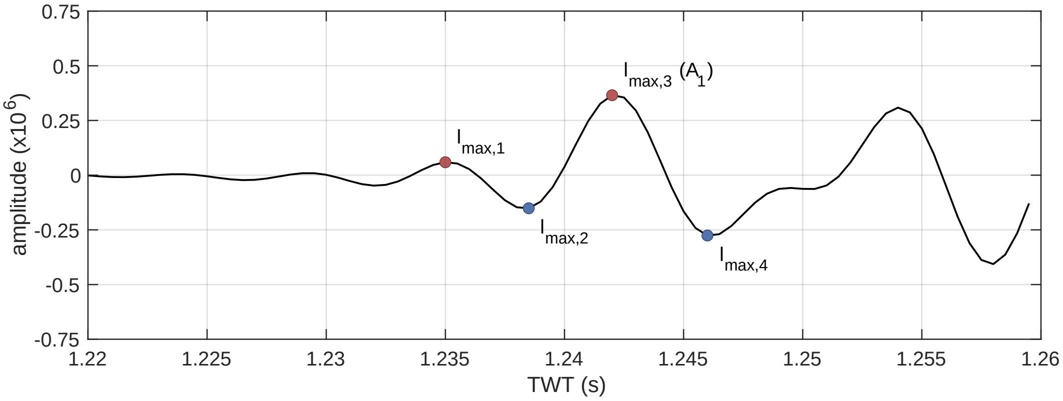

Supplementary Figure 15: **Amplitude analysis of the basal return.** Amplitude profile of basal return (A_1) of CMP 5361 in the along-flow profile. The dots show the identified local maxima $l_{\mathrm{max},1}$, $l_{\mathrm{max},2}$, $l_{\mathrm{max},3}$ (A_1) and $l_{\mathrm{max},4}$.

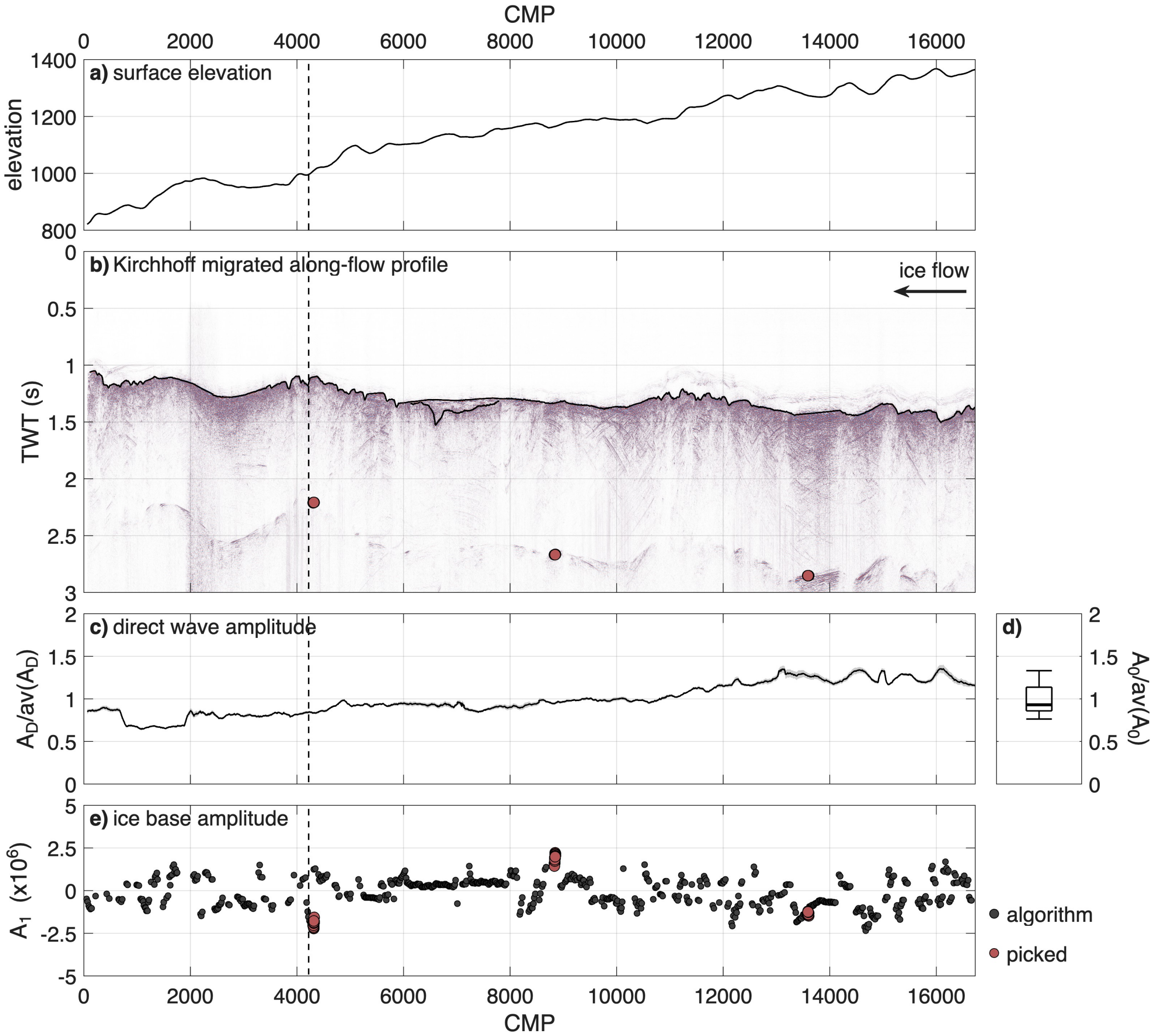

Supplementary Figure 16: **Amplitude analysis of the along-flow profile.** **a** Surface elevation. **b** Kirchhoff migrated vibroseismic section as a function of TWT and CMP. **c** Relative amplitude of direct wave (A_D). **d** Relative source amplitude (A_D). **e** Algorithm detected ice base amplitude (A_1). The vertical dashed line marks the crossing to profile 2024551.

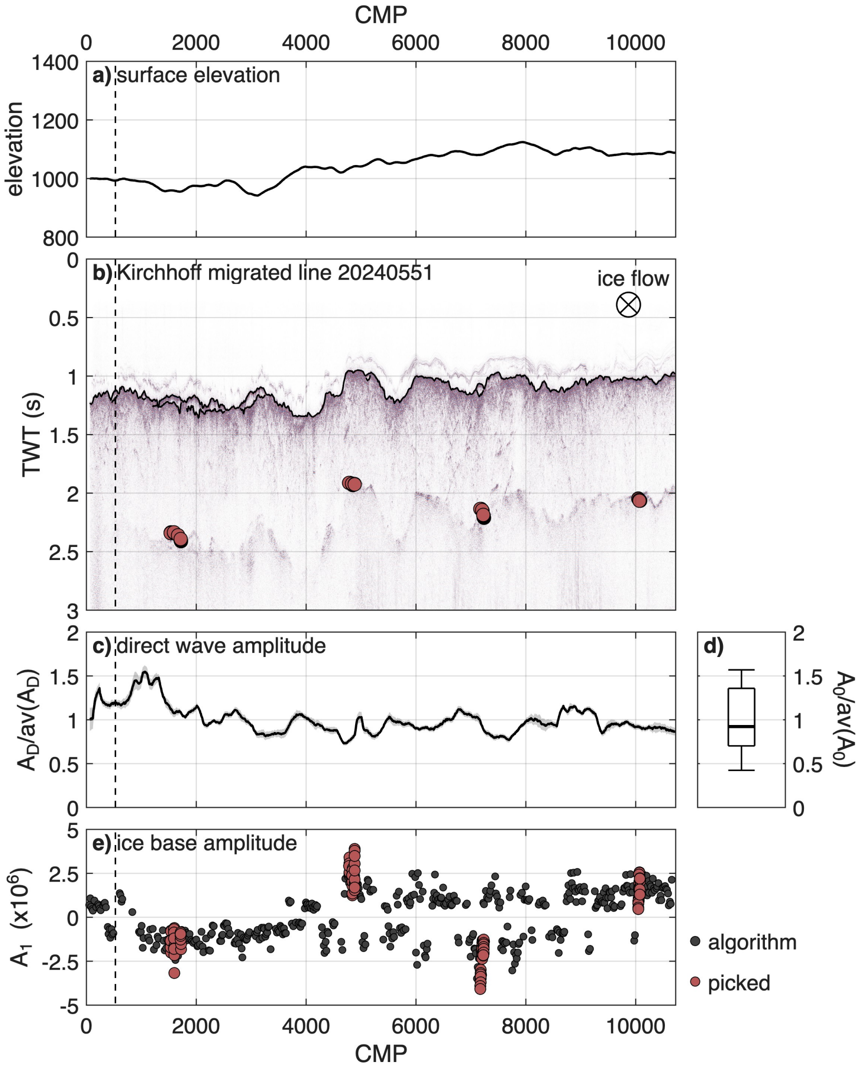

Supplementary Figure 17: **Amplitude analysis of the across-flow profile.** **a** Surface elevation. **b** Kirchhoff migrated vibroseismic section as a function of TWT and CDP. **c** Relative amplitude of direct wave (A_D). **d** Relative source amplitude (A_D). **e** Algorithm detected ice base amplitude (A_1). The vertical dashed line marks the crossing to profile 2023551.

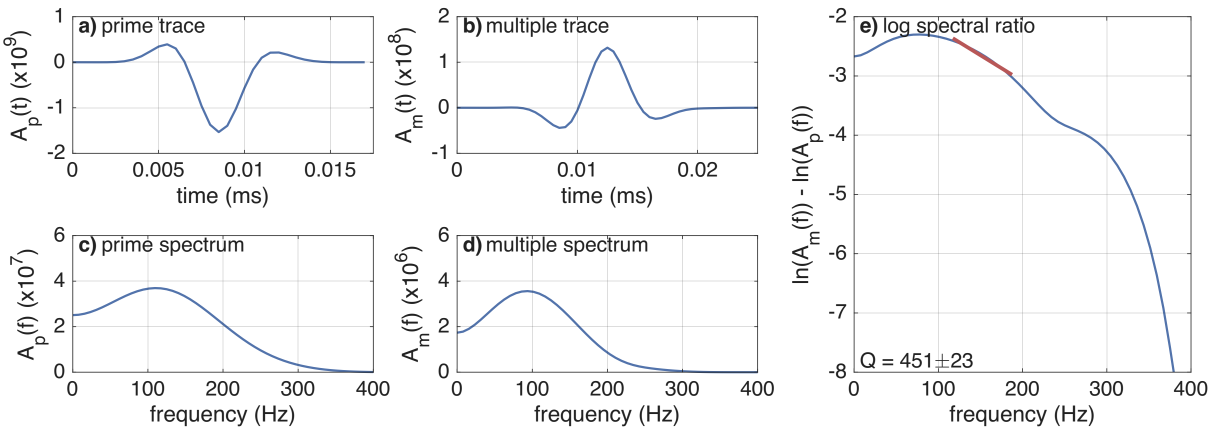

Supplementary Figure 18: **Quality factor of the along-flow profile. a, b** Stacked trace of prime (**a**) and multiple (**b**) basal reflection from 75 shots. **c, d** Stacked amplitude spectrum of prime (**c**) and multiple (**d**) basal reflection. **e** Log spectral ratio with gradient (red line) for a selected frequency range of 120–220 Hz and derived quality factor Q.

Members of participating consortium

The ITGC GHOST Team (thwaitesglacier.org/projects/ghost)

Ole Zeising[1], Olaf Eisen[1,2], Coen Hofstede[1], Ronan Agnew[3], Alex Brisbourne[3], Andrew O. Hoffman[4,5], Sridhar Anandakrishnan[6], Richard Alley[6], Rob Arthern[3], Robert G. Bingham[7], Julien Bodart[7,8,9], Louise Borthwick[10], Elizabeth Case[11,12], Knut Christianson[13], Kevin Hank[3], Nicholas Holschuh[14], Siobhan Killingbeck[15], Jonny Kingslake[16,11], Bernd Kulessa[15,17], Sierra Melton[6], Atsuhiro Muto[10], Felipe Napoleoni[7], Helen Ockenden[7,18], Rebecca Pearce[15,19], Byron R. Parizek[6,20], Kiya Riverman[21], Rebecca Schlegel[22], Emily Schwans[6], Andy Smith[3], Leigh A. Stearns[23], Nathan Stevens[6], Amanda Willet[6], Paul Winberry[24], Jaiden Zak[25], and Luke Zoet[25]

[1]Glaciology, Alfred Wegener Institute Helmholtz Centre for Polar and Marine Research, Bremerhaven, Germany

[2]Faculty of Geosciences, University of Bremen, Bremen, Germany

[3]Ice Dynamics and Palaeoclimate, British Antarctic Survey, Natural Environment Research Council, Cambridge, UK

[4]Department of Earth, Environmental, and Planetary Science, Rice University, Houston, TX, USA

[5]Lamont-Doherty Earth Observatory, Columbia University, Palisades, NY, USA

[6]Department of Geosciences, and Earth and Environmental Systems Institute, Pennsylvania State University, University Park, PA, USA

[7]School of GeoSciences, University of Edinburgh, Edinburgh, UK

[8]now at Climate and Environmental Physics, Physics Institute, University of Bern, Bern, Switzerland

[9]now at Oeschger Centre for Climate Change Research, University of Bern, Bern, Switzerland

[10]Department of Earth and Environmental Science, Temple University, Philadelphia, PA, USA

[11]Lamont-Doherty Earth Observatory, Columbia University, Palisades, NY, USA

[12]now at Institute of Marine and Atmospheric Research Utrecht, University of Utrecht, Utrecht, Netherlands

[13]Department of Earth and Space Sciences, University of Washington, Seattle, WA, USA

[14]Department of Geology, Amherst College, Amherst, MA, USA

[15]Faculty of Science and Engineering, Swansea University, Swansea, Wales, UK

[16]Department of Earth and Environmental Science, Columbia University, New York, NY, USA

[17]School of Natural Sciences – Physics, University of Tasmania, Hobart, Australia

[18]now at L'Institut des Géosciences de l'Environnement, Université Grenoble-Alpes, Saint-Martin-d'Hères, France
[19]now at Cascade Institute Royal Roads, University Colwood, BC, Canada
[20]Mathematics and Geoscience, Pennsylvania State University, DuBois, PA, USA
[21]College of Earth, Ocean, and Atmospheric Sciences, Oregon State University, Corvallis, OR, USA
[22]Department of Geosciences, University of Tübingen, Tübingen, Germany
[23]Department of Earth & Environmental Sciences, University of Pennsylvania, Philadelphia, PA, USA
[24]Department of Geological Sciences, Central Washington University, Ellensburg, WA, USA
[25]Department of Geoscience, University of Wisconsin-Madison, Madison, WI, USA

Supplementary References

[1] Holland, C. W. & Anandakrishnan, S. Subglacial seismic reflection strategies when source amplitude and medium attenuation are poorly known. *Journal of Glaciology* **55**, 931–937 (2009).

[2] Teng, T.-L. Attenuation of body waves and the Q structure of the mantle. *Journal of Geophysical Research (1896-1977)* **73**, 2195–2208 (1968).

[3] Båth, M. *Developments in Solid Earth Geophysics: Spectral Analysis in Geophysics* (Elsevier Scientific Publishing Company, 1974).

[4] Christianson, K. *et al.* Dilatant till facilitates ice-stream flow in northeast Greenland. *Earth and Planetary Science Letters* **401**, 57–69 (2014).

[5] Pritchard, H. D. *et al.* BEDMAP3 - Ice thickness, bed and surface elevation for Antarctica - gridding products (Version 1.0). NERC EDS UK Polar Data Centre [data set] (2024).

[6] Pritchard, H. D. *et al.* Bedmap3 updated ice bed, surface and thickness gridded datasets for Antarctica. *Scientific Data* **12**, 414 (2025).

[7] Holschuh, N., Christianson, K., Paden, J., Alley, R. & Anandakrishnan, S. Linking postglacial landscapes to glacier dynamics using swath radar at Thwaites Glacier, Antarctica. *Geology* **48**, 268–272 (2020).

[8] Morlighem, M. MEaSUREs BedMachine Antarctica, Version 2. Boulder, Colorado USA. NASA National Snow and Ice Data Center Distributed Active Archive Center, Accessed 12 April 2021 (2020).

[9] Morlighem, M. *et al.* Deep glacial troughs and stabilizing ridges unveiled beneath the margins of the Antarctic ice sheet. *Nature Geoscience* **13**, 132–137 (2020).

[10] Höyns, L.-S. *et al.* Improved basal drag of the West Antarctic Ice Sheet from L-curve analysis of inverse models utilizing subglacial hydrology simulations. *The Cryosphere* **19**, 2133–2158 (2025).
