# Supplementary material for: Hard rocks and deep wetlands beneath Thwaites Glacier in Antarctica
Source: Commun Earth Environ. 2026 Apr 24;7(1):366. doi: 10.1038/s43247-026-03502-2 (PMC13109048; doi:10.1038/s43247-026-03502-2)
Supplement: Supplementary file 3 — Description of Additional Supplementary Files [file 43247_2026_3502_MOESM3_ESM.pdf]

Description of Additional Supplementary Files

File name: Supplementary Movie 1

Description: Three-dimensional animation of Thwaites Glacier showing the vibroseismic profiles acquired in this study. The animation provides a three-dimensional perspective of the glacier. Surface elevation and bed topography are from the Bedmap3 dataset (Pritchard et al., 2025), and surface ice-flow velocities are from the MEaSUREs dataset (Mouginot et al., 2019).
